# Effectiveness of early pharmaceutical interventions in symptomatic COVID-19 patients: A randomized clinical trial

**DOI:** 10.12669/pjms.40.5.8757

**Published:** 2024

**Authors:** Shehnoor Azhar, Javed Akram, Waqas Latif, Naomi Cano Ibanez, Samiullah Mumtaz, Ali Rafi, Usman Aftab, Somia Iqtadar, Muhammad Shahzad, Fibhaa Syed, Bilal Zafar, Nighat Fatima, Saleh Saadat Afridi, Shehla Javed Akram, Muhammad Afzal Chaudhary, Farah Sadiq, Saifullah Goraya, Muhammad Hanif, Verda Ashraf, Saadia Ashraf, Humaira Akram, Tanwir Khaliq

**Affiliations:** 1 Shehnoor Azhar (BDS, MPH) Doctoral candidate in Epidemiology and Public Health, University of Granada; 2 Javed Akram, (MBBS, FRCP) Professor of Medicine and former Vice Chancellor, University of Health Sciences (UHS) Lahore; 3 Waqas Latif, (M.Phil Statistics) Data Analyst, University of Health Sciences (UHS) Lahore; 4 Naomi Cano Ibanez, (PhD) Department of Preventive Medicine and Public Health, University of Granada; 5 Samiullah Mumtaz, (MBBS, FCPS) Assistant Professor at Department of Medicine, King Edward Medical University (KEMU) Lahore; 6 Ali Rafi, (MBBS, MPhil) Faculty member at Department of Pharmacology, University of Health Sciences (UHS) Lahore; 7 Usman Aftab, (PhD) Assistant Professor at Department of Pharmacology, University of Health Sciences (UHS) Lahore; 8 Somia Iqtadar, (MBBS, FRCP) Associate Professor at Department of Medicine, King Edward Medical University (KEMU) Lahore; 9 Muhammad Shahzad, (PhD) Professor and Head of Department of Pharmacology, University of Health Sciences (UHS) Lahore; 10 Fibhaa Syed, (MBBS, FCPS) Assistant Professor at Department of Medicine, Shaheed Zulfiqar Ali Bhutto Medical, University (SZABMU) Islamabad; 11 Bilal Zafar, (MBBS) Registrar at Department of Medicine in HFH Rawalpindi, Rawalpindi Medical University (RMU) and affiliated hospitals; 12 Nighat Fatima, (MBBS) Medical Officer at Department of Medicine, Sargodha Medical College and its affiliated District Headquarter Hospital (DHQ) Sargodha; 13 Saleh Saadat Afridi, (MBBS, FCPS) Senior Registrar at Department of Medicine, Naseer Teaching Hospital (NTH) Peshawar; 14 Shehla Javed Akram, (MBBS, DTM&H, DCH, PhD candidate) Chief Executive Officer, Akram Medical Complex (AMC) Lahore; 15 Muhammad Afzal Chaudhary, (MBBS, FCPS) Associate Professor at Department of Medicine, Aziz Bhatti Shaheed Teaching Hospital (ABSTH) Gujrat; 16 Farah Sadiq , (MBBS, FCPS) Associate Professor of Medicine, Lahore General Hospital (LGH) Lahore; 17 Saifullah Goraya, (MBBS, FCPS) Professor of Medicine, Sargodha Medical College and its affiliated District Headquarter Hospital (DHQ) Sargodha; 18 Muhammad Hanif, (MBBS, FCPS) Professor of Medicine, Faisalabad Medical University (FMU) Faisalabad; 19 Verda Ashraf, (MBBS) Registrar at Department of Radiology, Akram Medical Complex (AMC) Lahore; 20 Saadia Ashraf, (MBBS, FCPS) Professor of Pulmonology, Khyber Teaching Hospital (KTH) Peshawar; 21 Humaira Akram, (MBBS, FCPS) Professor of Gynecology, Sargodha Medical College and its affiliated District Headquarter Hospital (DHQ) Sargodha; 22 Tanwir Khaliq, (MBBS, FRCS) Professor of Surgery and Vice Chancellor SZABMU Islamabad, Shaheed Zulfiqar Ali Bhutto Medical, University (SZABMU) Islamabad

**Keywords:** COVID-19, SARS-CoV-2, Effectiveness, Hospitalization, Follow-up, Patients

## Abstract

**Objective:**

We assessed the effectiveness of oral Hydroxychloroquine (HC), Azithromycin (AZ) and Oseltamivir (OS), alone or combined, among patients hospitalized with mildly symptomatic coronavirus infectious disease (COVID-19).

**Methods:**

Following the approval of the National Bioethics Committee and prospective registration (clinicaltrials.gov NCT04338698), a multicenter randomized clinical trial of adaptive design was conducted at 10 multispecialty hospitals in Pakistan. Patients were randomized into seven treatment groups. Starting April 15, 2020, consenting, eligible, otherwise healthy adult patients or those with co-morbidities under control, were recruited if they presented with mildly symptomatic COVID-19 (scored 3 on a 7-point ordinal scale anchored between 1 = not hospitalized, able to undertake normal activities, to 7 = death) confirmed by quantitative Real-Time Polymerase Chain Reaction (qRT-PCR). Two primary outcomes were assessed by day seven: Turning qRT-PCR negative; and clinical improvement of two points from the baseline. Outcome rates were compared using a chi-square test. Multiple imputations were applied to handle missing data. An interim data analysis was carried out on July 19, 2020, following which the study continued without treatment group changes. Data Safety and Monitoring Board advised to stop recruitment due to its futility on January 18, 2021.

**Results:**

Of 471 patients randomized, a total of 426 (90.4%) completed the follow-up for primary outcomes. Based on imputed data analyses at day seven: Total qRT-PCR negative cases were 137/471 (29%, 95% CI 25.0 - 33.4). By day seven, a total of 111/471 (23.5%, 95% CI 19.8 - 27.6) showed clinical improvement. No serious or non-serious adverse event was reported.

**Conclusions:**

Among patients with mild COVID-19, there was no statistically significant difference in the effectiveness of oral antimalarial, antiviral, or antibiotic treatments.

***Clinicaltrials.gov ID:*** NCT04338698

## INTRODUCTION

On March 11, 2020, severe acute respiratory syndrome of novel coronavirus (SARS-CoV-2) was declared a major global health emergency.^1^-^3^ This pandemic put low-and-middle-income countries (LMICs) at particular risk as they historically lacked an evidence-based culture to inform clinical care and prevention.^4^ In LMIC, the projected adverse impact of coronavirus infectious disease or COVID-19 was large. It was potentially containable if the disease progression could be stopped during its earliest phases through an affordable and readily available treatment.^5^ It was critical to optimize the limited hospital resources for more seriously ill patients and those with life-threatening conditions.^6^

In early 2020, many randomized clinical trials (RCTs) were launched to evaluate the various therapies for COVID-19.^7^ This was a scenario suitable for RCT of adaptive design since it was a new disease about which little was previously known.^8^-^11^ The RCTs investigated drugs like antibiotics, antiviral, anti-inflammatory, and immunotherapies. Scientific interest in immunomodulatory and anti-inflammatory potential of antimalarial like Hydroxychloroquine (HC) and Chloroquine skyrocketed to the extent that every fifth RCT included them as investigational drugs between February to July 2020.^8^,^12^ In March 2020, drug regulators in high-income countries like China, USA, and Ireland authorized their compassionate use for different stages of COVID-19 in hospitals. The decision was aimed at settings where RCT or participation in RCTs was not possible.^8^ So RCTs were much needed to inform the treatment guidelines worldwide, a situation that shaped the pandemic response in LMICs.^7^,^13^

While trials were ongoing, evidence syntheses using unpublished data went ahead considering the need to inform ongoing practice. Systematic reviews cited ongoing RCT comparisons of three drugs of wider interest at that time - HC, Azithromycin (AZ) and Oseltamivir (OS)^8^,^12^ AZ is an antibiotic (Macrolide); and OS is an antiviral that acts as a neuraminidase inhibitor.^14^,^15^ One of the reviews reported on mortality associated with HC or Chloroquine to treat patients with COVID-19.^8^ Half of the literature (14 of 28 included RCTs) it collated was unpublished at the time; 8 of 14 unpublished RCTs were still recruiting patients. Two of these RCTs used adaptive design.^16^,^17^ The publication of such RCTs will update pharmacological profiles for current indications of use of the drugs evaluated besides informing new drug development.^18^

We evaluated the effectiveness of the abovementioned three drugs, HC, AZ, and OS, individually and in various combinations, in treating mildly symptomatic COVID-19 hospitalized patients who had newly tested positive on Quantitative Real Time Polymerase Chain Reaction (qRT-PCR). In assessing the clearance of qRT-PCR and progression of milder COVID-19 upon administration of these drugs in a resource-deficient setting, this clinical trial sought to generate valuable evidence for early treatment of a previously unknown disease.

## METHODS

Approvals were granted by Ethics Review Committees at all participating sites and the National Bioethics Committee of Pakistan between the periods starting March 30, 2020, to May 4, 2020 (Annex-1). Prospective registration was completed with clinicaltrials.gov identification code - NCT04338698 dated April 8, 2020. The structured summary of this study was published in August 2020^16^ and its protocol was published in March 2022.^19^ This manuscript was prepared under Consolidated Standards of Reporting Trials (CONSORT) guidelines (checklist available as Annex-2).

### Trial design:

An adaptive design, set within a comprehensive cohort study was chosen because it permitted flexibility in a fast-changing clinical and public health scenario. This randomized study was multicenter, multiarm, multistage, with a parallel design.^20^ Of the total 15 multispecialty hospitals in eight cities invited to take part, recruitment took place at ten (Annex-1). All study investigators (hospital physicians) were trained to collect and report data electronically. The randomization sequence was created by the study biostatistician using Sealed Envelope Ltd 2019 in the allocation ratio of 1:1 for each of the seven treatment groups.

It was stratified by age groups (above or below 60 years) using random block sizes of 28 and 42 for the seven treatment arms. Computer-based randomization ensured concealment of allocation sequence. Before randomizing, recruiting physician confirmed eligibility and informed consent of the participant. Upon entering participant’s demographic details in the online dashboard, a unique patient ID for the said center and treatment arm was allocated automatically meaning the recruiting physician had no control over assigning intervention. Once intervention group was allocated, both physician and patients knew the medication given.

### Participants:

Hospitalized individuals over 18 years of age, who newly tested positive on qRT-PCR with mild COVID-19 symptoms (fever, dry cough, myalgia) were eligible if they were either otherwise healthy without a known comorbidity (particularly diabetes mellitus and hypertension) or if those comorbid condition(s) was/were controlled by daily medication(s). Pregnant or lactating women, those already on COVID-19 treatment, under respiratory distress or severely dyspneic or under intensive care, immunocompromised, suspected or known cases of cardiac failure, liver, or kidney diseases, were excluded. Each participant underwent baseline investigations at the time of enrollment including liver function tests, renal function tests, urinalysis, and complete blood count. A tobacco smoker was defined as self-reported smoking of at least 10 cigarettes a day.

### Interventions:

Participants were randomized into seven intervention groups (A to G) while the eighth group (H) comprised individuals who declined randomizing but consented to be observed during their stay at the hospital. Groups A (HC), B (OS) and C (AZ) received single drug; D (HC + OS), E (HC + AZ), F (OS + AZ) were given three different combinations of two drugs, while G was administered all three study drugs (HC + AZ + OS). HC was given 200 mg orally 8hr thrice a day for five days; OS given 75 mg orally twice a day for five days; and AZ given 500 mg orally daily on day one, followed by 250 mg orally twice a day on days 2-5. All drugs were packaged under label NOT FOR SALE from the source. Soon after the onset of pandemic, Food and Drug Administration (FDA) issued Emergency Use Authorization (EUA) for compassionate use of Chloroquine and Hydroxychloroquine or HC for COVID-19.^21^ Following the announcement, several countries restricted their export hence raising concerns for global supply of these drugs.^22^ So, we investigated only Hydroxychloroquine in this study because of its widespread local production and availability unlike Chloroquine that ran in short supply.

### Outcomes:

An ear-nose-throat trained nurse collected participants’ nasopharyngeal and oropharyngeal swab samples on alternate days till day 7 that could be extended up to day 14 post-hospital admission, while the physician recorded the clinical status at each participating site. Results of qRT-PCR tests were reportable either as positive or negative or without viral load estimation (as initially envisaged). The physician reported data (qRT-PCR positive or negative, and clinical status on 7-point ordinal scale) via electronic dashboard hosted at the principal site. The scale comprised of the following: 1- Not hospitalized, able to resume normal activities, 2- Not hospitalized, but unable to resume normal activities, 3- Hospitalization, not requiring supplemental oxygen, 4- Hospitalization, requiring supplemental oxygen, 5- Hospitalization, requiring noninvasive mechanical ventilation, 6- Hospitalization, requiring invasive mechanical ventilation, 7-Death.^23^

### Data collection:

Participants scored 03 on the abovementioned scale at the time of randomizing. All baseline investigations at the time of enrollment and the subsequent clinical data were recorded by investigators in an electronic case reporting form (eCRF). Neither paper-based reporting nor its archiving was possible considering the risk of cross-contamination throughout the period of data collection. In case of positive test and/or no clinical improvements by Day seven, drug administration and data collection continued till Day 14. As previously envisaged, qRT-PCR reporting based on viral load was not possible since local laboratories only reported the status as positive or negative. No confirmatory or second test was performed in case of a negative result. A standardized PCR diagnostic platform could not be used in this study.

### Oversight:

Data Safety and Monitoring Board (DSMB) was notified by Drug Regulatory Authority of Pakistan (notification available in Annex-3). Each DSMB member is listed under the acknowledgement section. All study investigators were trained to monitor and report via eCRF any serious Adverse Drug Reaction (sADR) or Adverse Events (AEs). If suspected, both sADR and AEs were to be assessed by the site investigators for meeting the reporting criteria. Upon being reported to the Principal Investigators and subsequent confirmation, all sADR and AEs were reportable to ethics committees, DSMB, and patient(s), via study website. Following the announcement by Solidarity trial of World Health Organization (WHO) to review the Hydroxychloroquine/Chloroquine arms suspecting cardiotoxicity among participants^8^, Principal Investigators voluntarily reached out to the DSMB to review the study data collected thus far dated July 19, 2020. No additional risk was found, and the recruitment to the trial continued without any changes to the design (correspondence available in Annex-3). On January 19, 2021, DSMB advised that recruitment should be ended due to its futility.

### Statistical analyses:

The parameters for formal sample size calculation in a new disease of a previously unknown virus were not available. The final sample size was to be subjected to periodic reviews at each stage of the adaptive design by the DSMB.^16^,^19^ The recruitment target of 520 participants as mentioned in the protocol was not met.^19^ The study data were analyzed by biostatistician based at the University of Health Sciences, Lahore, Pakistan, in line with prospective registration and the clinical trial protocol.^16^ Interim analysis was conducted with intention to treat without disclosing groups to the trial investigators dated July 19, 2020, maintaining confidentiality in interpretation of interim results. Stata 16 (College Station, TX) was primarily used for analyses. The seven-point ordinal scale for recording clinical condition of participants was converted into binary defining clinical improvement as lowering of score of two points from the baseline compared. Chi-square statistics were computed for both primary outcomes on day seven along with 95% CI and p-values. Additional analyses of the two outcomes were reported on day-14. Patient data was considered missing if he/she withdrew informed consent or did not have both outcomes recorded on eCRF on day seven. Annex-4 includes anonymized dataset, statistical output, and codes of all analyses with multiple imputations for missing data as well as the original or unimputed data.^24^,^25^

### Patient and public involvement:

Neither members of the public nor patients were involved in administrative, scientific, or academic activities related to this trial. To contribute towards public health education on COVID-19 in general and specifically on the evidence-based use of the study drugs, this trial was promoted on all multimedia platforms (broadcast, digital, documentary, and social media) on non-commercial basis. Interim results were publicly announced in an academic seminar dated July 23, 2020. The study outcome on clinical improvement or recovery from disease was among those included in a core outcome set for COVID-19 developed with patient involvement.^26^

We also complied with the GRIPP-2 reporting checklist (Annex-5). A documentary on this trial was made available on free social media platforms (The Making of PROTECT: A Randomized Clinical Trial - https://www.youtube.com/watch?v=37MZRuHsq24) to sustain public interest in other clinical trials seeking to discover COVID-19 treatment(s). A website theprotect.com.pk was also launched to share the study milestones and updates relevant to SARS-CoV-2. The web address of this site was mainly promoted via the website of University of Health Sciences Lahore.

## RESULTS

**Fig.1:**
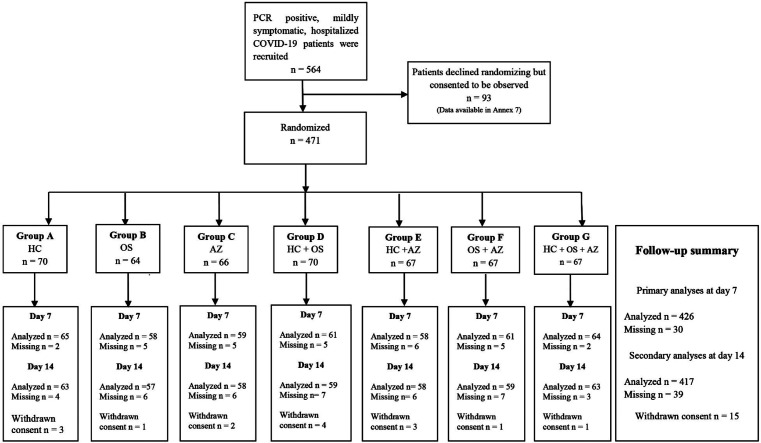
Flow chart of Pakistan Randomized and Observational Trial to Evaluate Coronavirus Treatment (PROTECT) with follow-up summary (HC Hydroxychloroquine Phosphate, OS Oseltamivir, AZ Azithromycin).

A total of 471 hospitalized patients were randomized between April and November 2020. Of the 45 missing on day seven, 30 patients were lost to follow-up while 15 withdrew consent to participate further after randomization (Fig.1). Table-I summarizes demographic characteristics of those randomized. The demographic data of 93 patients who consented only to be observed during hospitalization are presented in Annex-6. Randomized and non-randomized patients did not significantly differ in demographic characteristics. 59.6% of study participants were males, their age in years ranged from 18-94 with mean and standard deviation (M + SD) 42.6 + 16.3. About 41% reported earning more than PKR 50,000/- ($312.5) a month. About 8.5% were diabetic, 4.7% hypertensive, and 5.1% had coexisting diabetes and hypertension. Self-reported tobacco smoking defined as at least 10 cigarettes a day was 4.6%. Only 12 participants had a history of foreign travel within a week prior to positive PCR test (statistics not tabulated).

**Table-I:** Baseline demographic characteristics of participants of Pakistan Randomized and Observational Trial to Evaluate Coronavirus Treatment (PROTECT).

Characteristics n (%)	A (HC) 70 (14.8)	B (OS) 64 (13.6)	C (AZ) 66 (14.0)	Groups n (%) D (HC + OS) 70 (14.8)	E (HC + AZ) 67 (14.2)	F (OS + AZ) 67 (14.2)	G (HC + OS + AZ) 67 (14.2)	Total N = 471 (100)
Age in years presented as	42.4 ± 16.7	42.4 ± 14.8	40.8 ± 17.0	42.7 ± 15.8	43.1 ± 17.1	42.8 ± 17.5	43.5 ± 15.4	42.6 ± 16.3
Mean ± Standard Deviation Range	18 – 80	18 - 86	18 - 88	18 – 83	18 - 80	18 - 94	18 – 80	18 - 94
Sex								
Male	43 (61.4)	45 (70.3)	42 (63.6)	41 (58.5)	36 (53.7)	42 (62.6)	32 (47.7)	281 (59.6)
***Marital Status***								
Married	59 (84.2)	50 (78.1)	48 (72.7)	56 (80)	51 (76.1)	49 (73.1)	56 (83.5)	369 (78.3)
Household*								
Single family in a house	38 (54.2)	34 (53.1)	37 (56.0)	35 (50.0)	33 (49.2)	33 (49.2)	35 (52.2)	245 (52.0)
Monthly Family Income PKR**								
≤ 25,000/-	14 (20.0)	19 (29.6)	26 (39.3)	19 (27.1)	19 (28.3)	24 (35.8)	24 (35.8)	145 (30.8)
> 25000 < 50000	23 (32.8)	18 (28.1)	19 (28.7)	11 (15.7)	21 (31.3)	20 (29.8)	19 (28.3)	131 (27.8)
≥ 50000	33 (47.1)	27 (42.1)	21 (31.8)	40 (57.1)	27 (40.2)	23 (34.3)	24 (35.8)	195 (41.4)
***Comorbidities***								
Diabetes	5 (7.1)	6 (9.3)	4 (6.0)	7 (10.0)	9 (13.4)	1 (1.4)	8 (12.0)	40 (8.5)
Hypertension	4 (5.7)	2 (3.1)	1 (1.5)	3 (4.2)	2 (2.9)	5 (7.4)	5 (7.4)	22 (4.7)
Both diabetes and hypertension coexisted	4 (5.7)	2 (3.1)	7 (6.0)	2 (2.8)	4 (5.9)	2 (2.9)	3 (4.4)	24 (5.1)
Tobacco smoking	4 (5.7)	3 (4.6)	6 (9.0)	3 (4.2)	2 (2.9)	2 (2.9)	2 (3.0)	22 (4.6)

* Whether living as single family per household or multiple families sharing a house,

** 1 United States Dollar ~ 160 Pakistan Rupees (on April 30, 2020) HC = Hydroxychloroquine, OS = Oseltamivir, AZ = Azithromycin. Statistics presented as number and percentages in parentheses unless otherwise stated.

The results for the primary outcomes at day seven for both imputed and original data are summarized in Table-II. The total qRT-PCR negative cases were 137/471 (29%, 95% CI 25.0 - 33.4) with group breakdown being: A: 19/70 (27.1%, 95% CI 17.1 - 39.0); B: 20/64 (31.2%, 95% CI 20.2 - 44.0); C: 18/66 (27.7%, 95% CI 17.0 - 39.6); D: 15/70 (21.4%, 95% CI 12.5 - 32.8); E: 18/67 (26.8%, 95% CI 16.7 - 39.0); F: 17/67 (25.3%, 95% CI 15.5 - 37.4); and G: 30/67 (44.8%, 95% CI 32.6 - 57.4) (p=0.08). By day 7, a total of 111 (23.5%, 95% CI 19.8 - 27.6) showed clinical improvement with group breakdown being: A: 15/70 (21.4%, 95% CI 12.5 - 32.8); B: 13/64 (20.3%, 95% CI 11.2 - 32.2); C: 12/66 (18.1%, 95% CI 9.7 - 29.6); D: 19/70 (27.1%, 95% CI 17.1 - 39.0); E: 18/67 (26.8%, 95% CI 16.7 - 39.0); F: 14/67 (20.8%, 95% CI 11.9 - 32.5); and G: 20/67 (29.8%, 95% CI 19.2 - 42.2) (p=0.64).

**Table-II:** Primary outcomes of participants of Pakistan Randomized and Observational Trial to Evaluate Coronavirus Treatment (PROTECT).

Outcomes at hospitalization day 7 (imputed data)	A (HC) n = 70 (14.8)	B (OS) n = 64 (13.6)	C (AZ) n = 66 (14.0)	Groups D (HC + OS) n = 70 (14.8)	E (HC + AZ) n = 67 (14.2)	F (OS + AZ) n = 67 (14.2)	G (HC + OS + AZ) n = 67 (14.2)	p-value	Total N = 471
PCR negative n (%)	19 (27.1%)	20 (31.2%)	18 (27.7%)	15 (21.4%)	18 (26.8%)	17 (25.3%)	30 (44.8%)	0.08	137 (29.0)
(95% CI)	(17.1 – 39.0)	(20.2 – 44.0)	(17.0 – 39.6)	(12.5 – 32.8)	(16.7 – 39.0)	(15.5 – 37.4)	(32.6 – 57.4)		(25.0 – 33.4)
Clinical improvement n (%)	15 (21.4)	13 (20.3)	12 (18.1)	19 (27.1)	18 (26.8)	14 (20.8)	20 (29.8)		111 (23.5)
(95% CI)	(12.5 – 32.8)	(11.2 – 32.2)	(9.7 – 29.6)	(17.1 – 39.0)	(16.7 – 39.0)	(11.9 – 32.5)	(19.2 – 42.2)	0.64	(19.8 – 27.6)
Outcomes at hospitalization day 7 (original data)	A (HC) n = 65	B (OS) n = 58	C (AZ) n = 59	D (HC + OS) n = 61	E (HC + AZ)	F (OS + AZ)	G (HC + OS + AZ)	p-value	Total N = 426
	(15.2)	(13.6)	(13.8)	(14.3)	n = 58 (13.6)	n = 61 (14.3)	n = 64 (15.0)		
PCR negative n (%)	18 (27.6%)	19 (32.7%)	17 (28.8%)	12 (19.6%)	16 (27.5%)	16 (26.2%)	29 (45.3%)	0.08	127 (29.8)
(95% CI)	(17.3 – 40.1)	(21.0 – 46.3)	(17.7 – 42.0)	(10.5 – 31.8)	(16.6 – 40.8)	(15.7 – 39.)	(32.8 – 58.2)		(25.5 – 34.4)
Clinical improvement n (%)	13 (20.0)	12 (20.7)	10 (16.9)	13 (21.3)	13 (22.4)	11 (18.03)	19 (29.7)		91 (21.3)
(95% CI)	(11.1 – 31.7)	(11.1 – 33.3)	(8.4 – 28.9)	(11.8 – 33.6)	(12.5 – 35.2)	(9.3 – 29.9)	(18.9 – 42.4)	0.69	(17.5 – 25.5)

Multiple imputations were carried out for missing data. All participants were recruited at score 3 on the seven-category ordinal scale. Clinical improvement defined as lowering of score of two points from the baseline.

Table-III summarizes the secondary analyses - study outcomes on day 14. Laboratory results of 54 patients and clinical data of 57 patients were missing. Results were separately tabulated for both imputed and original data. It showed the total qRT-PCR negative cases were 342/471 (72.6%, 95% CI 68.3 - 76.5) with group breakdown being: A: 55/70 (78.5%, 95% CI 67.1 - 87.4); B: 39/64 (60.94%, 95% CI 47.9 - 72.8); C: 49/66 (74.2%, 95% CI 61.9 - 84.2); D: 45/70 (64.2%, 95% CI 51.9 - 75.3); E: 51/67 (76.1%, 95% CI 64.1 - 85.6); F: 46/67 (68.6%, 95% CI 56.1 - 79.4); and G: 57/67 (85.0%, 95% CI 74.2 - 92.6) (p=0.02). By day 14, a total of 196 (41.6%, 95% CI 37.1 - 46.2) showed clinical improvement with group breakdown being: A: 23/70 (31.8%, 95% CI 22.0 - 45.1); B: 25/64 (39.0%, 95% CI 27.1 - 52.0); C: 30/66 (45.4%, 95% CI 33.1 - 58.1); D: 29/70 (41.4%, 95% CI 29.7 - 53.8); E: 33/67 (49.2%, 95% CI 36.8 - 61.7); F: 24/67 (35.8%, 95% CI 24.4 - 48.4); and G: 32/67 (47.7%, 95% CI 35.4 - 60.3) (p=0.38).

**Table-III:** Secondary outcomes of participants of Pakistan Randomized and Observational Trial to Evaluate Coronavirus Treatment (PROTECT).

Outcomes at hospitalization day 14 (imputed data)	A (HC) n = 70 (14.8)	B (OS) n = 64 (13.6)	C (AZ) n = 66 (14.0)	D (HC + OS) n = 70 (14.8)	Groups E (HC + AZ) n = 67 (14.2)	F (OS + AZ) * n = 67 (14.2)	G (HC + OS + AZ) n = 67 (14.2)	p-value	Total N = 471
PCR negative n (%)	55 (78.5)	39 (60.9)	49 (74.2)	45 (64.2)	51 (76.1)	46 (68.6)	57 (85.0)	0.02	342 (72.6)
(95% CI)	(67.1 – 87.4)	(47.9 – 72.8)	(61.9 – 84.2)	(51.9 – 75.3)	(64.1 – 85.6)	(56.1 – 79.4)	(74.2 – 92.6)		(68.3 – 76.5)
Clinical improvement n (%)	23 (32.8)	25 (39.0)	30 (45.4)	29 (41.4)	33 (49.2)	24 (35.8)	32 (47.7)		196 (41.6)
(95% CI)	(22.0 – 45.1)	(27.1 – 52.0)	(33.1 – 58.1)	(29.7 – 53.8)	(36.8 – 61.7)	(24.4 – 48.4)	(35.4 – 60.3)	0.38	(37.1 – 46.2)
Outcomes at hospitalization day 14 (original data)	A (HC)	B (OS)	C (AZ)	D (HC + OS)	E (HC + AZ)	F (OS + AZ)	G (HC + OS + AZ)	p-value	Total N = 417
	n = 63 (15.1)	n = 57 (13.6)	n = 58 (13.9)	n = 59 (14.1)	n = 58 (13.9)	n = 59* (14.1)	n = 63 (15.1)		
PCR negative n (%)	49 (77.7)	38 (66.6)	43 (74.1)	38 (64.4)	44 (75.8)	41 (69.4)	55 (87.3)	0.07	308 (73.8)
(95% CI)	(65.5 – 87.2)	(52.9 – 78.5)	(60.9 – 84.7)	(50.8 – 76.4)	(62.8 – 86.1)	(56.1 – 80.8)	(76.5 – 94.3)		(69.3 – 78.0)
Clinical improvement n (%)	20 (31.75)	24 (42.1)	25 (43.1)	22 (37.2)	25 (43.1)	18* (32.4)	31 (49.2)		165 (39.8)
(95% CI)	(20.5 – 44.6)	(29.1 – 55.9)	(30.1 – 56.7)	(25.0 – 50.8)	(30.1 – 56.7)	(20.0 – 45.9)	(36.3 – 62.1)	0.40	(35.1 – 44.7)

Multiple imputations were carried out for missing data All participants were recruited at score 3 on the seven-category ordinal scale. Clinical improvement defined as lowering of score of two points from the baseline

* Group F: Compared to outcome 1 (PCR negative), outcome 2 (clinical improvement) data of 3 additional patients in group F was missing hence n = 56 and total N = 414.

Of the total 16 deaths reported up to two weeks of hospital stay, 14/426 (3.7%) occurred in first week with group breakdown being: A: 1/65 (1.5%); B: 2/58 (3.4%); C: 6/59 (10.1%); D: no deaths in 61 patients; E: 2/58 (3.4%); F: 2/61 (3.2%); and G: 1/64 (1.5%). Mortality in HC and HC combination groups (A, D, E, G) was 4/248 (1.61%). These results update to the mortality data of this study shared in July 2020 with the collaborative meta-analysis aimed at assessing mortality associated with the use of HC in RCTs for COVID-19.^8^ No serious or non-serious adverse event was reported.

## DISCUSSION

This multicenter RCT was undertaken as part of a broader national effort to mitigate the impact of a potentially fatal pandemic in a resource-limited setting. None of the investigated drugs namely HC, AZ, and OS, alone or in combination, were found to be effective in treating mildly symptomatic COVID-19 hospitalized patients who had newly tested positive on qRT-PCR. For outcomes capturing the progression of mild COVID-19, no statistically significant differences were found among seven treatment groups in LMIC setting. The data on mortality, a core COVID-19 outcome, were updated.

Our study represented an evidence-based response to SARS-CoV-2 in addition to various administrative measures referred to as non-pharmaceutical measures introduced worldwide to break the chain of coronavirus transmission at the outset of pandemic.^27^ Without any evidence of therapeutic benefit against COVID-19, demand of antimalarials like HC and antibiotics like AZ had peaked early in 2020 stoking fears for supply shortages even for their indicated use.^8^ Its coverage on various media platforms promoted the evidence-based healthcare delivery at the outset of the pandemic. Despite limited experience of conducting multicenter RCTs as a strategy to discover treatment of a rapidly spreading new disease, patients belonging to a variety of sociocultural backgrounds were recruited across 10 hospitals.

Drafting of this manuscript was guided by CONSORT (Annex-2) and GRIPP-2 (Annex-5) checklists. The standard RCT design tends to be rigid, something that was not really an option in early 2020 for a previously unknown disease in a fast-changing public health scenario.^10^,^11^ The adaptive design permitted head-to-head comparison of three drugs alone and in various combinations comprising seven treatment groups with the added flexibility of the ability to re-evaluate and change the comparisons during the study.^10^,^11^ The trial could eliminate or add treatments as the circumstances developed and new evidence emerged, based on the independent input of the DSMB. This assessment took place on one occasion with the interim review being done on July 19, 2020, following concerns associated with the use of HC in World Health Organization (WHO) SOLIDARITY trial.^28^

COVID-19 research also highlighted the risk to integrity of investigations and the need for transparency in reporting. In this regard, a recently published international consensus statement on clinical trials has been a significant contribution in addition to previous guidelines such as Trustworthiness in Randomised Controlled Trials or TRACT.^29^,^30^ The reporting of this study was aligned with this framework to set a benchmark for future conduct of clinical trials in similar settings. We assessed this manuscript under TRACT to demonstrate compliance with integrity and transparency principles (Annex-5 contains the completed checklist).

A *priori* sample size estimation for this study was challenging since no reliable estimates of COVID-19 treatment effect sizes had existed, unlike other known diseases. The final number of patients recruited was to be decided based on the advice of the DSMB. The sample size mentioned in the study registration, structured summary, and protocol was only indicative.^16^,^19^ It was not achieved because it became difficult to recruit hospitalize patients at a time when data on ineffectiveness of study drugs was widely being reported.^8^ As the burden of severe COVID-19 symptoms grew, hospitals prioritized admitting such patients and shifting those with milder symptoms either to other health facilities or advising isolation at home.

Resultantly, the study investigators had limited access to recruiting patients as per criteria. Lower than expected recruitment limited the study power and our inability to demonstrate statistically significant differences between seven treatment groups could be the result of type-II error. The width of observed Confidence Intervals (CIs) reported in results above might have been another consequence of lower sample size. Concurrently, trials for COVID-19 vaccines started worldwide including study centers in Pakistan during September 2020 that made recruitment for drug trials further challenging.

There existed various logistical constraints and paucity of resources that prevented use of standardized testing kit and laboratory equipment in this study, compared with many other well-resourced studies.^4^ Resultantly, many different testing kits were used across 10 study centers. With more resources, it would have been possible to carry out additional clinical and diagnostic investigations measures of COVID-19 pathophysiology (chest radiographs, and serological markers like C-Reactive Proteins and D-dimers). Observed together with primary outcomes, such data could be useful in understanding prognostic factors particularly when very little was known on disease progression.^31^

WHO recommends publishing results of interventional clinical trial within 24 months of completion of data collection.^32^ Many studies that begun during the same period published their findings much earlier than this RCT.^8^,^33^ Key team members of our study group prioritized vaccine trials for COVID-19 contributing to the delay in publication.^34^,^35^ However, the finding remains relevant today as they contribute to updating of pharmacological profiles of the drugs evaluated and add support to deployment of RCTs of adaptive designs as effective drug discovery tools in a future pandemic.^18^

Our study also complemented a global health strategy to discover pharmaceutical treatments that could stop progression of COVID-19 to a more severe forms right at the outset of the pandemic. Of the hundreds of RCTs in 2020 that evaluated dozens of pharmaceuticals and biologic products (immunoglobulins, herbal compounds and supplements, and cell therapies) worldwide, this RCT was among the 3% that compared HC and AZ head-to-head with an antiviral like OS besides combinations of these three drugs.^12^ Early literature on effectiveness of HC in COVID-19 was informed by two global RCTs - WHO SOLIDARITY and RECOVERY.^8^ Compared to 65 patients given HC in relatively lower dosage in our study and mortality of 1.5%, HC arms of these two trials recruited 947 and 1561 patients with mortality of around 11% and 27%, respectively.^8^ In 2021, a meta-analysis of 14 published RCTs including the two larger ones (mentioned above) had collectively reported 14.5% mortality among a total of 3712 patients (14.57%) given HC.^8^

Only 12 of the 471 (2.5%) participants reported a history of foreign travel within a month preceding COVID-19 qRT-PCR compared with up to 8% being reported in studies with much higher sample size.^36^ More broadly, our findings are in line with those reported by now that HC has no clinical effectiveness in COVID-19.^13^,^14^

Several studies have documented adverse unintended consequences of non-pharmaceutical interventions (NPIs) in LMICs amid an overall lack of health emergency.^27^ Within LMICs, socioeconomically disadvantaged communities were impacted disproportionately in losing livelihoods, poor mental health outcomes, and likelihood of coronavirus infection on being forced to stay in-doors within relatively smaller yet crowded households. In recruiting hospitalized patients and ensuring mass media coverage, this study promoted a science-based public health approach aiming to reduce overreliance on NPIs. Our RCT demonstrated the capacity of trials to build sustainable scientific linkages embedded within clinical practice to generate evidence while providing care.^6^ In utilizing supply chains of local manufacturers of drugs to deliver to various study sites, a feasible model of academic-industry linkage was built that could be replicated in future.^37^,^38^ Health experts and local policymakers could build further on this experience and develop flexible strategies while preparing for future public health emergencies.^39^

While several LMICs have opted to innovate and improved their research credentials during SARS-CoV-2,^37^,^40^ sustaining it would require better understanding of Good Clinical Practices (GCP) under the given sociocultural context.^41^ Future scholarship under health emergencies should focus on addressing the various barriers and facilitators to adoption and interconnectedness of GCP at institutional, legislative, and regulatory tiers.^42^ Such a framework will likely underpin a research culture most able to deliver an RCT of adaptive design when it is most needed in a future pandemic. Ethics committees and institutional review boards should leverage the potential of RCTs of adaptive designs by encouraging their greater use in line with the corresponding integrity statements.^29^

## CONCLUSION

Among patients with mild COVID-19, there was no statistically significant difference in the effectiveness of oral antimalarial, antiviral, or antibiotic treatments. This study demonstrated the feasibility of offering therapeutic options while conducting a timely pragmatic trial in a low-and-middle-income country at the onset of pandemic.

**Table:** 

Center name (City)	Name of EC/IRB*	Reference number (Date of approval)
University Of Health Sciences (UHS) Constituent Hospital (Gujranwala)	Ethics Review Committee	UHS/REG-20/ERC/858 (March 30, 2020)
Akram Medical Complex (Lahore)	IRB-AMC	2020-4-10-Admin/CT (March 30, 2020)
King Edward Medical University (Lahore)	Institutional Review Board	267/RC/KEMU (April 14, 2020)
Lahore General Hospital (Lahore)	Notified endorsement of NBC via Principal	Misc./27251/LGH (April 25, 2020)
Shaheed Zulfiqar Ali Bhutto Medical University (Islamabad)	Ethics Review Board	F.1-1/2015/ERB/SZAMBU/550 (April 4, 2020)
Rawalpindi Medical University (Rawalpindi)	Institutional Research Forum	35/IREF/RMU/220 (April 14, 2020)
Faisalabad Medical University (Faisalabad)	Notified endorsement of approvals by NBC** and UHS via Vice Chancellor	5461/FMU (April 24, 2020)
Sargodha Medical College (Sargodha)	Notified endorsement of approvals by NBC and UHS via Administration	SU/Admin(HR)/1749-55 (May 4, 2020)
Khyber Teaching Hospital (Peshawar)	Institution Research & Ethics Board	836/ADR/KMC (April 23, 2020)
Nawaz Sharif Teaching Hospital (Gujrat)	Notified endorsement of NBC via Principal	(MC)(PN)01/20 (May 4, 2020)

* (Ethics Committee/Institutional Review Boards) and

** (National Bioethics Committee).
